# Alzheimer’s disease prevention: from risk factors to early intervention

**DOI:** 10.1186/s13195-017-0297-z

**Published:** 2017-09-12

**Authors:** Marta Crous-Bou, Carolina Minguillón, Nina Gramunt, José Luis Molinuevo

**Affiliations:** 1 Barcelonaβeta Brain Research Center—Pasqual Maragall Foundation, C/Wellington, 30, 08005 Barcelona, Spain; 2 CIBER Fragilidad y Envejecimiento Saludable (CIBERFES), Madrid, Spain

**Keywords:** Alzheimer’s disease, Amyloid beta, Prevention, Risk factors, Susceptibility, Early intervention, Clinical trials

## Abstract

Due to the progressive aging of the population, Alzheimer’s disease (AD) is becoming a healthcare burden of epidemic proportions for which there is currently no cure. Disappointing results from clinical trials performed in mild–moderate AD dementia combined with clear epidemiological evidence on AD risk factors are contributing to the development of primary prevention initiatives. In addition, the characterization of the long asymptomatic stage of AD is allowing the development of intervention studies and secondary prevention programmes on asymptomatic at-risk individuals, before substantial irreversible neuronal dysfunction and loss have occurred, an approach that emerges as highly relevant.

In this manuscript, we review current strategies for AD prevention, from primary prevention strategies based on identifying risk factors and risk reduction, to secondary prevention initiatives based on the early detection of the pathophysiological hallmarks and intervention at the preclinical stage of the disease. Firstly, we summarize the evidence on several AD risk factors, which are the rationale for the establishment of primary prevention programmes as well as revising current primary prevention strategies. Secondly, we review the development of public–private partnerships for disease prevention that aim to characterize the AD continuum as well as serving as platforms for secondary prevention trials. Finally, we summarize currently ongoing clinical trials recruiting participants with preclinical AD or a higher risk for the onset of AD-related cognitive impairment.

The growing body of research on the risk factors for AD and its preclinical stage is favouring the development of AD prevention programmes that, by delaying the onset of Alzheimer’s dementia for only a few years, would have a huge impact on public health.

## Background

The prevalence of dementia worldwide is estimated to be over 45 million people [1] and is predicted to triple by 2050 as a consequence of increased life expectancy, establishing dementia as one of the biggest global public health challenges. Alzheimer’s disease (AD) is the most common form of dementia and accounts for 60–80% of cases [1]. AD is a progressive neurodegenerative disease, irreversible and disabling, causing a large socioeconomic burden [2].

The criteria for AD diagnosis have been revised extensively, and experts agree that the hallmark pathological criteria include increased levels of amyloid-beta (Aβ) peptide, which is deposited extracellularly in diffuse and neuritic plaques, and hyperphosphorylated tau (p-tau), a microtubule assembly protein that accumulates intracellularly as neurofibrillary tangles [3]. Initial diagnostic efforts focused on patients at the dementia stage of the disease and, only recently, the importance of a long pre-dementia stage, preceding the clinical onset of the disease symptoms, has been recognized. As the disease progresses, the subject’s cognition changes from an initial phase where it is fully preserved to a final stage characterized by dementia [4]. The initial silent and asymptomatic stage, referred to as preclinical AD, is characterized by a sequence of pathophysiological hallmarks that start to appear about 20 years before the onset of symptoms [5].

Unfortunately, none of the drugs tested to date in clinical trials in order to change the course of the disease have shown effective results in AD dementia [6]. Therefore, many interventional studies are currently moving their focus to cognitively healthy individuals at risk of developing AD (before substantial irreversible neuronal network dysfunction and loss, associated with overt clinical symptoms, have occurred) as the best strategy to reduce AD incidence and prevalence.

In this review, we will summarize current strategies for AD prevention, from primary prevention strategies based on identifying risk factors and risk reduction, to secondary prevention based on early detection of the pathophysiological hallmarks and intervention at the preclinical stage. Furthermore, we will discuss a number of selected environmental risk factors for AD, and we will describe currently ongoing interventional initiatives focused on primary prevention of AD, as well as some of the public–private partnerships (PPPs) for disease prevention that are setting up a framework to identify and select individuals for clinical trials focused on preclinical stages.

## Primary prevention

### Modifiable risk factors for AD

Since AD develops over a long preclinical stage that can last for several decades, the extent to which risk factors assessed in late life or shortly before the onset of clinical symptoms are a result of pathological changes rather than having a causal relationship has been discussed intensively. Longitudinal studies that include participants in early mid-life have been crucial to assess the relationship between early or mid-life exposures and cognitive decline or AD later in life [7].

Observational studies have identified several modifiable risk factors for AD. Based on a comprehensive systematic review of the evidence related to risk factors for cognitive decline and AD, the US National Institutes of Health highlighted diabetes mellitus, smoking, depression, mental inactivity, physical inactivity and poor diet as being associated with increased risk of cognitive decline, AD, or both [8]. Later on, this list was further extended to include hypertension, obesity and low educational attainment [9]. Recently, an association was demonstrated between the presence of vascular risk factors in mid-life and amyloid deposition later in life [10], even though some of these factors are still under debate. It has been estimated that up to a third of AD cases are potentially attributed to these factors and, consequently, could be prevented [11].

Modifiable risk factors for AD are mostly related to either cardiovascular risk factors (diabetes, hypertension and obesity) or lifestyle habits (e.g. smoking, physical activity, diet, mental and social activity). Diabetes has been associated with an increased risk of AD (RR = 1.39). It has been suggested that diabetes could increase the risk by directly affecting Aβ accumulation in the brain since hyperinsulinemia disrupts brain Aβ clearance by competing for the insulin-degrading enzyme [12, 13]. In contrast, other studies suggest that diabetes might increase the risk of cerebrovascular but not AD pathology, and that at least part of the relationship between diabetes and cognitive impairment may be modified by neuropathology [14]. Even though the association between high blood pressure and AD risk is complex and age related, evidence suggest that mid-life, and not late-life, hypertension is associated with a 50% increased risk of AD and dementia in later life. Elevated blood pressure might increase the risk of AD by decreasing the vascular integrity of the blood–brain barrier, resulting in protein extravasation into brain tissue, which can consequently lead to cell damage, apoptosis and an increase in Aβ accumulation [15]. However, the direction of a possible causal relationship between hypertension and subsequent cognitive decline is under debate since there is also increasing evidence that hypertension may be a protective response to cerebral hypoperfusion, which is demonstrable 10 years prior to AD onset [16, 17]. Observational studies have shown a U-shaped relationship between weight and cognitive performance: both low and high body weight have been associated with increased risk of AD and cognitive impairment. This association might also have an age-dependent component. Data also exist for reverse causation in the years preceding disease onset; that is, loss of body weight might be caused by cognitive impairment during the pre-dementia phase of AD. Consequently, the relationship between body weight and AD seems to be a consequence of mid-life obesity, which could increase the risk of AD by 60%. Even though the underlying mechanisms of this association remain unknown, studies suggest that insulin resistance and co-incidence with diabetes mellitus may play a role [18].

With regards to lifestyle-related factors, the association between smoking and AD risk has been controversial and remains unclear. Most observational studies show an association between current smoking and increased risk of dementia, AD and cognitive decline. Even though relative risks for AD are relatively small (RR = 1.20–1.60), nearly 14% of AD cases are estimated to be potentially attributable to smoking due to its high prevalence [9]. Smoking may increase AD risk through several mechanisms, mostly related to oxidative stress and inflammatory responses [19]. Epidemiological studies have shown that physical activity has a beneficial effect on brain health, which could be explained through multiple mechanisms including activation of brain plasticity, promotion of brain vascularization, stimulation of neurogenesis, reduction of inflammation levels or even by decreasing the rate of amyloid plaque formation. In comparison with sedentary behaviours, individuals with high levels of physical activity have been shown to reduce their AD risk by half (RR = 0.72 for all causes of dementia; RR = 0.55 for AD) [20, 21]. Involvement in exercise programmes has been shown to significantly improve cognitive function in healthy older people. In contrast, physical inactivity has been associated with an increased risk of cognitive impairment in most longitudinal studies. However, whilst there is convincing evidence of an association between physical activity and subsequent cognitive decline, the direction of a possible causal relationship is still debatable. In their recent study, Sabia et al. [22] did not find evidence of a neuroprotective effect of physical activity and suggest that previous findings showing a lower risk of dementia in physically active people may be attributable to reverse causation (i.e. due to a decline in physical activity levels in the preclinical phase of dementia). The Mediterranean diet (MD) [23] may also protect against cognitive decline, AD and all-cause dementia [24, 25]. Observational studies have shown that higher adherence to a MD is associated with slower rates of cognitive decline, reduced progression to AD and improvements in cognitive function. Specifically, it has been shown that adherence to a MD might have a beneficial effect on memory, executive function and visual constructs [25]. A recent intervention study, PREDIMED, has shown that a MD supplemented with olive oil or nuts is associated with improved cognitive function [26].

Cognitive, social and intellectual activity jointly with higher education and occupational attainment have been shown to decrease risk of cognitive decline and dementia by increasing cognitive reserve, the capacity of the brain to resist the effects of neuropathological damage [27]. Observational studies consistently show that people who engage in mentally stimulating activities are less likely to develop AD (RR = 0.54). About 19% of AD cases worldwide are potentially attributable to low education attainment, making it the risk factor that contributes to the largest proportion of AD cases [9]. Helping to build a cognitive reserve that enables individuals to continue functioning at a normal level despite experiencing neurodegenerative changes seems to have a high impact on disease onset. The beneficial impact of bilingualism on brain reserve and consequently on AD risk and cognition has been highlighted recently [28, 29]. Studies suggest that lifelong bilingualism may delay the onset of dementia by about 4.5 years by contributing to cognitive reserve and, consequently, protecting against neurodegeneration.

The fact that a third of AD cases are potentially attributable to modifiable risk factors highlights the potential of risk factor reduction for disease prevention. However, the need for therapeutic strategies for the remaining two-thirds of cases is still urgent.

### Primary prevention strategies

Intervention strategies focused on modifiable risk factors for the disease are becoming a realistic and relevant therapeutic strategy for disease prevention. Interestingly, large epidemiological cohort studies suggest that the incidence of age-specific dementia is decreasing, probably due to a better control of cardiovascular risk factors [30, 31].

As an example, several intervention studies focused on primary prevention of dementias are currently ongoing, mainly in Europe, with the aim of reducing disease incidence. It is worth mentioning the Finnish Geriatric Intervention Study to Prevent Cognitive Impairment and Disability (FINGER) study [32] which aimed to investigate whether a multidomain intervention could prevent cognitive decline among older people. Additionally, investigators aimed to assess the effect of this multidomain intervention on disability, quality of life, depressive symptoms, the use of health care services and vascular risk factors. The 1200 participants of the FINGER study had an increased risk of cognitive decline. To date, results from this large, long-term, randomized controlled trial (RCT) have demonstrated that a multidomain intervention including diet, exercise, cognitive training and monitoring vascular risk can improve or maintain cognitive functioning in older people (60–77 years old) from the general population at risk of dementia [33].

The Prevention of Vascular Dementia by Intensive Care (PreDIVA) trial aimed to determine whether the control of cardiovascular risk factors could reduce dementia incidence. PreDIVA tested whether a multicomponent intervention targeting vascular risk factors could prevent new cases of dementia [34]. Researchers conducted a 6-year, open cluster RCT in primary care with over 3500 cognitively healthy participants aged 70–78. Results showed that this multidomain intervention focused on vascular care did not result in a reduced incidence of all-cause dementia in an unselected population of older people and did not have an effect on mortality, cardiovascular disease or disability, despite a greater improvement in systolic blood pressure in the intervention group compared with the control. Investigators suggested that the absence of effect might have been caused by modest baseline cardiovascular risks and high standards of usual care received by the control group. Hence, these results do not rule out the potential benefit of a better management of cardiovascular risk factors on brain health [35].

Another relevant study is the Multidomain Alzheimer Preventive Trial (MAPT) which aimed to evaluate the efficacy of a single multidomain intervention including nutritional counselling, physical exercise and cognitive training, the efficacy of an isolated omega 3 fatty acid supplementation and the efficacy of a combination of the two previously mentioned interventions on the prevention of cognitive decline in frail older participants aged 70 years or older [36]. A total of 1680 participants were followed for a period of 3 years; the study also collected imaging and biological data to be potentially used in future AD prevention and treatment trials. Both the multidomain intervention and polyunsaturated fatty acids, either individually or in combination, had no significant effects on cognitive decline over 3 years in older people with memory complaints. Particularly, the fact that participants had subjective memory complaints at enrolment, their mean age was 75 years and that almost half of participants showed a clinical dementia rating of 0.5 might have been important methodological limitations of the study. It might have been too late for the preventive intervention to show its potential efficacy. However, a post-hoc analysis performed on those participants with a positive amyloid scan showed a significant benefit in favour of the intervention [37].

Recently, a new study based on an eHealth intervention has been presented. Healthy Ageing Through Internet Counselling in the Elderly (HATICE) aims to investigate whether a multidomain intervention to optimize self-management of lifestyle-related risk factors for cardiovascular disease in older individuals, delivered through a coach-supported interactive platform, can improve the cardiovascular risk profile and reduce the risk of cardiovascular disease and cognitive decline [38]. The study has recruited 2600 people older than 65 years at increased risk of cardiovascular disease. Investigators developed an intuitive, easy-to-use platform, allowing for widespread use among older adults with only limited computer skills. Results from this study are expected in 2017.

Based on experiences and data from the intervention studies on AD prevention described (summarized in Table 1), investigators from the European Dementia Prevention Initiative (EPDI) have recently launched the Multimodal Preventive Trials for AD (MIND-AD) project. While all trials described previously are testing the effects of multimodal interventions targeting vascular, dietary and lifestyle-related risk factors in older adults not suffering with dementia, the aim of the MIND-AD project is to identify effective prevention strategies for AD and dementia tailored to different “at-risk” groups. The novel approach of this project consists of multidomain interventions, inclusion of novel models of delivery (e.g. computer-based cognitive training, medical food), critical feedback from trial participants and synergistic use of data from several European countries with over 10,000 participants. Furthermore, a pilot study in which a multimodal preventive intervention will be tested for the first time in prodromal AD will be conducted (http://www.mind-ad.eu/).

**Table 1:** Description of randomized controlled trials of multidomain interventions for prevention of cognitive impairment, dementia or AD

	FINGER	PreDIVA	MAPT	HATICE
Study population	1260 community dwellers from previous population-based non-intervention studies	3526 community dwellers	1680 community dwellers	2600 community dwellers
Main inclusion criteria	CAIDE Dementia Risk Score ≥ i6 and cognitive performance at the mean level or slightly lower than expected for age assessed with the Consortium to Establish a Registry for Alzheimer’s Disease neuropsychological battery	All community-dwelling older people without dementia registered with a participating general practice	Frail older people: spontaneous memory complaint, limitation in one instrumental activity of daily living and slow walking speed (i.e. lower than 0.8 m/s)	Older adults without dementia at increased risk of cardiovascular disease
Age at enrolment	60–77 years	70–78 years	≥70 years	≥65 years
Study design	Multicentre, randomized controlled trial	Multicentre, cluster randomized controlled trial	Multicentre, randomized controlled trial	Multicentre, randomized controlled trial
Intervention	Multidomain: (1) nutritional guidance; (2) physical exercise; (3) cognitive training and social activity; and (4) intensive monitoring and management of metabolic and vascular risk factors	Multidomain: (1) nutritional advice; (2) physical activity advice; and (3) vascular care including medical treatment of risk factors	Multidomain: (1) nutritional advice; (2) physical activity advice; (3) cognitive training; and (4) vascular care, and/or 800 mg docosahexaenoic acid per day	e-health: multidomain interactive Internet platform, stimulating self-management of vascular risk factors, with remote support
Control group	General health advice	Usual care	Placebo alone	Static Internet platform with basic health information
Duration	2 years plus 5-year follow-up	6 years	3 years plus 2-year follow-up	18 months
Outcomes	Primary: change in cognitive performance. Secondary: dementia; disability; depression; vascular risk factors and outcomes; quality of life; utilization of health resources; and neuroimaging biomarkers.	Primary: cumulative incidence of dementia and disability score (ALDS) at 6 years of follow-up. Secondary: incident cardiovascular disease and cardiovascular and all-cause mortality. Other secondary: cognitive decline, depression, blood pressure, body mass index, blood lipid concentrations, and glucose concentration.	Primary: change in memory function. Secondary: cognitive performance, functional status, depression, cost-effectiveness	Primary: outcome is a composite score based on the average *z*-score of the difference between baseline and 18-month follow-up values of systolic blood pressure, low-density-lipoprotein and body mass index. Secondary: include the effect on the individual components of the primary outcome, the effect on lifestyle-related risk factors, incident cardiovascular disease, mortality, cognitive functioning, mood and cost-effectiveness
Status	Completed in 2014	Completed in 2015	Completed in 2014	Due to finish in 2017
Findings (if available)	The multidomain intervention could improve or maintain cognitive functioning [24]	The intervention did not result in a reduced incidence of all-cause dementia and did not have an effect on mortality, cardiovascular disease or disability [26]	The multidomain intervention and polyunsaturated fatty acids, either alone or in combination, had no significant effects on cognitive decline. Post-hoc data showed that the combination of polyunsaturated fatty acids and multidomain intervention had potential beneficial effects in participants with CAIDE scores ≥ 6 or higher or evidence of brain amyloid pathology, suggesting that people with increased risk of dementia might benefit most from the intervention [28]	N/A
Reference	[23]	[25]	[27]	[29]

*AD* Alzheimer’s disease, *ALDS *Academic Medical Center Linear Disability Score, *CAIDE* Cardiovascular Risk Factors, Aging, and Incidence of Dementia, *FINGER* Finnish Geriatric Intervention Study to Prevent Cognitive Impairment and Disability, *HATICE* Healthy Ageing Through Internet Counselling in the Elderly, *MAPT* Multidomain Alzheimer Preventive Trial, *PreDIVA* Prevention of Vascular Dementia by Intensive Care

In the USA, an example of a cognitive-related intervention is the ACTIVE study, the biggest RCT on cognitive training in healthy older adults [39]. The ACTIVE study included over 2800 cognitively healthy participants older than 65 years who attended 10 group sessions during a 6-week period where, according to the intervention arm, they received specific training in either memory, reasoning or speed of processing. A subgroup of participants in each arm received a few booster sessions just before the first and third years post intervention. The intervention groups were compared among them, and with a non-intervention control group. Results confirmed that domain-specific training was beneficial for maintaining cognition in the targeted domain. Two years later, the intervention showed modest benefits for cognitive training in cognitive performance, which were maintained at the 5-year follow-up [40], and slight benefits in self-reported function in daily living activities [41]. After the 10-year follow-up investigators concluded that those who received any intervention showed less functional decline in daily living activities, and those trained in reasoning and speed of processing also showed better performance in the targeted abilities [42]. Dementia rates at 10 years were significantly lower in participants in the speed of processing intervention group [43].

Results from intervention studies highlight the methodological limitations underlying the design and implementation of effective preventive strategies. Studies should take into account that AD is multifactorial and, consequently, interventions should be specific to risk profiles. Small long-term effects on cognition, as those shown by FINGER, are of high relevance for public health, since they may significantly contribute to the reduction of the overall burden of AD. An effective strategy for AD prevention could start with recommendations addressed to the general population (particularly to cognitively healthy subjects older than 50 years) on how to manage lifestyle and cardiovascular risk factors. In parallel, a multidomain long-term intervention could be offered to individuals identified as being at increased risk of developing AD (e.g. subjects with subjective memory concerns or a family history of dementia).

## Secondary prevention

New consensus diagnostic criteria for preclinical AD, together with the identification of at-risk individuals through the use of biomarkers that are altered before clinical decline (i.e. amyloid deposition in the brain), are key for identifying at-risk asymptomatic individuals who are ideal candidates to participate in secondary prevention trials. Cerebral Aβ deposition is considered a necessary, but not sufficient, step on the path towards AD development [44].

Furthermore, results from most trials focused on Aβ-centric approaches at the dementia stage of AD have been disappointing, suggesting that those participants have already surpassed the optimal therapeutic window for intervention [6]. The preclinical stage might offer the optimal window for therapeutic success and the opportunity to intervene at earlier stages of the continuum, arresting or delaying the onset of cognitive decline and ultimately dementia.

This approach is also supported by studies showing that biomarker abnormality in preclinical AD occurs in a temporal manner: low Aβ42 in cerebrospinal fluid (CSF) and cerebral amyloid deposits precede elevated CSF tau, topographical cerebral injury and cognitive decline [45, 46]. These pathologic changes may start decades before the onset of symptoms: in pre-symptomatic PSEN mutation carriers, CSF Aβ42 decline has been observed 25 years before clinical symptoms, whilst brain Aβ deposition and elevated CSF tau have been detected 15 years before symptom onset [47]. Observational studies have shown that cognitively normal individuals with abnormal levels of AD biomarkers exhibit longitudinal cognitive decline [48, 49], suggesting that they are at increased risk of progressing to cognitive impairment and, consequently, to dementia.

Several PPPs for disease prevention are currently ongoing, setting up a framework to identify and select individuals to be included in trials focused on the AD preclinical stage. These initiatives also aim to maximize efficiency to obtain a clinical signal and develop sensitive outcomes for detecting early decline, through new trial designs.

The Dominantly Inherited Alzheimer Network (DIAN) is an international PPP determined to understand a rare form of AD that is dominantly inherited, caused by a genetic mutation in presenilin 1 (PSEN1), presenilin 2 (PSEN2) or amyloid precursor protein (APP) [50]. The DIAN pioneers observational studies in pre-symptomatic individuals based on the hypothesis that understanding this form of AD may provide insight into the more common form of the disease. DIAN is enrolling participants who are adult children of a parent with a mutated gene known to cause dominantly inherited AD. Participants may or may not carry the gene, and may or may not have disease symptoms. The DIAN is developing an expanded registry to enable trials for research-neglected individuals such as familial early-onset AD, to increase the power for successful trials and to test more drugs. Moreover, the project supports studies related to autosomal dominant AD to increase the chance of success of treatment trials. The ultimate objective of DIAN is a successful prevention trial that yields the approval of the first disease-modifying drug, bolsters interest in developing improved drugs and demonstrates a clear pathway to prevent AD in the general population.

The Alzheimer’s Prevention Initiative (API) is an international collaborative initiative established to provide an innovative approach to Alzheimer’s prevention research by evaluating the most promising therapies in cognitively normal people who, based on their age and genetic background, are at the highest imminent risk of developing AD symptoms [51]. The API is focused on prevention and treatment trials, biomarker studies and registries, with the ultimate goal being to delay, reduce the risk or prevent AD clinical onset. The API has set up a robust registry where members receive regular informational materials including new trials.

A major European PPP, the European Prevention of Alzheimer’s Dementia (EPAD) project, funded by the Innovative Medicines Initiative, is designed to increase the likelihood of the successful development of new treatments for the secondary prevention of Alzheimer’s dementia by creating a novel environment for testing numerous interventions [52]. EPAD aims to create the necessary infrastructure, including a registry and a longitudinal cohort study, for delivering an adaptive trial for secondary prevention of AD. EPAD will test different agents in pre-dementia AD participants through an infrastructure providing: improvement of access to existing cohorts and registries; development of a registry of approximately 24,000 individuals who might be at increased risk of developing AD; establishment of a longitudinal cohort study of 6000 subjects; and establishment of an adaptive, proof-of-concept trial including 1500 participants at any given time.

In the USA, the Global Alzheimer’s Platform (GAP) has already been launched, while Canada and Japan are about to promote similar sister initiatives. There are several projects under the GAP umbrella: GAP-track is establishing a global standing, trial-ready platform to reduce clinical testing cycle times by 2 years or more and achieve greater efficiency and uniformity in trial populations through large, well-characterized trial-ready cohorts, certified clinical trial sites and an adaptive proof-of-concept trial mechanism. This will enable the delivery of efficient and effective proof-of-concept and confirmatory trials and ultimately a more rapid delivery of effective therapies to patients or persons at risk. GAP-net is creating a trial-ready network of sites all over the USA for the prevention and treatment of AD [53].

Secondary prevention trials in asymptomatic participants with preclinical AD who are amyloid positive are already ongoing. Some of these trials are summarized in Table 2. In the context of the dominantly inherited form of AD, the DIAN-TU trial targets cognitively normal individuals, participants with mild cognitive impairment or mild dementia who are either known to have an AD-causing mutation or at risk for such a mutation. The aim is to assess the efficacy of gantenerumab and solanezumab by determining whether treatment improves cognitive outcomes and disease-related biomarkers. The API-ADAD (for Autosomal Dominant AD) trial will recruit preclinical members of the *PSEN1 E280A* mutation carrier kindred. It will evaluate the efficacy of crenezumab in 200 cognitively normal individuals who carry the *PSEN1 E280A* mutation. The study will also include 100 *PSEN1 E280A* mutation non-carriers who will receive placebo only.

**Table 2:** Description of currently ongoing AD prevention clinical trials

	DIAN-TU	API-ADAD	A4	TOMMORROW	API-GENERATION	EARLY
Estimated enrolment	210	300	1150	3494	1340	1650
Target population/specific characteristics	Autosomal dominant Alzheimer’s disease (ADAD) mutation carriers or persons that have a 50% chance of carrying an ADAD mutation	Membership in PSEN1 E280A mutation carrier kindred	Cognitively normal, positive brain amyloid PET	Cognitively normal with genetic risk (*TOMM40* and *APOE* genotype)	Cognitively normal *APOE-*ε*4* homozygous	Cognitively normal positive amyloid
Age	18–80 years	30–60 years	65–85 years	65–83 years	60–75 years	60–85 years
Phase	Phase II/III	Phase II	Phase III	Phase III	Phase II/III	Phase II/III
Compound	Gantenerumab Solanezumab	Crenezumab	Solanezumab	Pioglitazone	CAD106 CNP520	JNJ-54861911
Mechanism	Anti-Aβ antibodies	Anti-Aβ antibody	Anti-Aβ antibody	PPAR-γ agonist	Aβ vaccine BACE inhibitor	BACE inhibitor
Status	Ongoing, not recruiting	Active, not recruiting	Recruiting	Active, not recruiting	Recruiting	Recruiting
Primary outcome	Composite Cognitive Test Score	Composite Cognitive Test Score	Composite Cognitive Test Score	Time to diagnosis of mild cognitive impairment (MCI) due to AD	Time to diagnosis of MCI or dementia due to AD Composite Cognitive Test Score	Composite Cognitive Test Score
Study duration	4 years	5 years	3 years	5 years	5 years	4.5 years
Trial identifier	NCT01760005	NCT01998841	NCT02008357	NCT01931566	NCT02565511	NCT02569398
Expected completion	December 2019	September 2020	October 2020	July 2019	August 2023	May 2023

Source: https://clinicaltrials.gov (accessed May 2017) [45]

*A4* Anti-Amyloid Treatment in Asymptomatic Alzheimer’s study, *A*β amyloid beta, *AD* Alzheimer’s disease, *API-ADAD* Alzheimer’s Prevention Initiative for Autosomal Dominant Alzheimer’s Disease, *API-GENERATION* A Study of CAD106 and CNP520 Versus Placebo in Participants at Risk for the Onset of Clinical Symptoms of Alzheimer's Disease, *BACE* Beta-secretase, *DIAN* Dominantly Inherited Alzheimer Network, *EARLY* An Efficacy and Safety Study of JNJ-54861911 in Participants Who Are Asymptomatic at Risk for Developing Alzheimer's Dementia, *PET* Positron Emission Tomography, *PPAR* Peroxisome proliferator activated receptor, *PSEN1* presenilin 1, *TOMMORROW* Biomarker Qualification for Risk of Mild Cognitive Impairment (MCI) Due to Alzheimer's Disease (AD) and Safety and Efficacy Evaluation of Pioglitazone in Delaying Its Onset

Regarding sporadic AD, the Anti-Amyloid Treatment in Asymptomatic Alzheimer’s (A4) study aims to test whether an anti-amyloid antibody, solanezumab, can slow cognitive loss caused by AD. The overall goal is to test whether solanezumab can help slow the cognitive loss associated with amyloid build-up. The study plans to enrol 1150 cognitively healthy adults with a positive amyloid scan who are randomly assigned to receive the investigational drug or placebo. New studies with a similar approach, such as the EARLY study, are currently ongoing. The purpose of EARLY is to evaluate whether treatment with the BACE inhibitor JNJ-54861911 slows cognitive decline, as measured by a composite cognitive measure, the Preclinical Alzheimer Cognitive Composite (PACC), in cognitively healthy amyloid-positive participants.

Other studies employ a different approach, selecting participants through a genetic risk profile. The TOMMORROW study will use a genetic risk assignment algorithm (BRAA) to determine the 5-year risk of developing MCI due to AD. The efficacy of low-dose pioglitazone to delay the onset of MCI due to AD will be tested in cognitively normal, high-risk individuals, as identified by the BRAA. The study uses *TOMM40* and *APOE* genotype and age to identify individuals who may be at a high or low risk of developing MCI in the following 5 years. High-risk individuals will be randomly assigned to the active or placebo arms. A small group of low-risk individuals will receive placebo. The API-GENERATION study, a randomized, double-blind, placebo-controlled, two-cohort parallel group study, aims to evaluate the efficacy of CAD106 and CNP520 in participants at risk for the onset of AD symptoms. The purpose is to determine the effects of each of the two therapies given separately, on cognition, global clinical status and underlying pathology. Cognitively unimpaired individuals, homozygotes for *APOE-*ε*4* aged 60–75 years, were selected as a high-risk population for progression to MCI due to AD and/or dementia.

## Conclusions

Epidemiological evidence of AD risk factors is contributing and encouraging the development of primary prevention initiatives. Current trials and strategies are necessary steps whose results are helping to improve future designs, bringing some post-hoc analysis on the potential benefits of risk factor reduction on disease incidence. Identifying individuals at risk of developing the disease might be the key to success of intervention studies.

Ongoing clinical trials in asymptomatic participants with either a positive amyloid biomarker or at increased genetic risk of AD will help ascertain whether secondary prevention initiatives are valid strategies and whether clinical trials of 3–5 years are sufficient for delaying cognitive decline, and consequently the onset of Alzheimer’s dementia.

The implementation of effective prevention strategies is not free from challenges since they require the identification, characterization and participation of asymptomatic individuals, developing new primary endpoints, implementing the use of AD biomarkers in cognitively healthy people, disclosing these results and performing long trials, whose optimal length is yet to be determined. The incorporation of biomarkers to identify individuals at risk of developing AD dementia is a key step for the identification of ideal candidates to participate in trials and secondary prevention initiatives.

Clinical trials focused on the preclinical stage of AD might help to maximize the possibility of obtaining a clinical signal as well as developing sensitive methods for detecting early decline through new trial designs.

## Abbreviations

A4: Anti-Amyloid Treatment in Asymptomatic Alzheimer’s study
AD: Alzheimer’s disease
API: Alzheimer’s Prevention Initiative
API-ADAD: Alzheimer’s Prevention Initiative for Autosomal Dominant Alzheimer’s Disease
Aβ: Amyloid beta
CSF: Cerebrospinal fluid
DIAN: Dominantly Inherited Alzheimer Network
EPAD: European Prevention of Alzheimer’s Dementia
FINGER: Finnish Geriatric Intervention Study to Prevent Cognitive Impairment and Disability
GAP: Global Alzheimer’s Prevention
HATICE: Healthy Ageing Through Internet Counselling in the Elderly
MAPT: Multidomain Alzheimer Preventive Trial
MD: Mediterranean diet
MIND-AD: Multimodal Preventive Trials for Alzheimer’s Disease
PPP: Public–private partnership
PreDIVA: Prevention of Vascular Dementia by Intensive Care
RCT: Randomized controlled trial
RR: Relative risk
